# Dyhidro-β-agarofurans natural and synthetic as acetylcholinesterase and COX inhibitors: interaction with the peripheral anionic site (AChE-PAS), and anti-inflammatory potentials

**DOI:** 10.1080/14756366.2022.2091554

**Published:** 2022-07-10

**Authors:** Julio Alarcón-Enos, Evelyn Muñoz-Núñez, Margarita Gutiérrez, Soledad Quiroz-Carreño, Edgar Pastene-Navarrete, Carlos Céspedes Acuña

**Affiliations:** a Departamento de Ciencias Básicas, Universidad del Bío-Bío, Chillán, Chile; b Instituto de Química, Universidad de Talca, Talca, Chile

**Keywords:** Acetylcholinesterase, butyrylcholinesterase, dihydro-β-agarofurans, COX-2, molecular docking, Alzheimer’s disease

## Abstract

In order to find molecules of natural origin with potential biological activities, we isolate and synthesise compounds with agarofuran skeletons (epoxyeudesmanes). From the seeds of *Maytenus disticha* and *Maytenus magellanica* we obtained six dihydro-β-agarofurans, and by means of the Robinson annulation reaction we synthesised five compounds with the same skeleton. The structures were established on the basis of NMR, IR, and MS. The evaluated compounds showed inhibitory activity on the acetylcholinesterase enzyme and on the COX enzymes. Compound **4** emerged as the most potent in the acetylcholinesterase inhibition assay with IC_50_ 17.0 ± 0.016 µM on acetylcholinesterase (AChE). The compounds evaluated were shown to be selective for AChE. The molecular docking, and the propidium displacement assay suggested that the compounds do not bind to the active site of the enzyme AChE, but rather bind to the peripheral anionic site (PAS) of the enzyme, on the other hand, the natural compound **8**, showed the best inhibitory activity on the COX-2 enzyme with an IC_50_ value of 0.04 ± 0.007 µM. The pharmacokinetic profile calculated in silico using the SWISSADME platform shows that these molecules could be considered as potential drugs for the treatment of neurodegenerative diseases such as AD.

## Graphical Abstract

**Figure:**
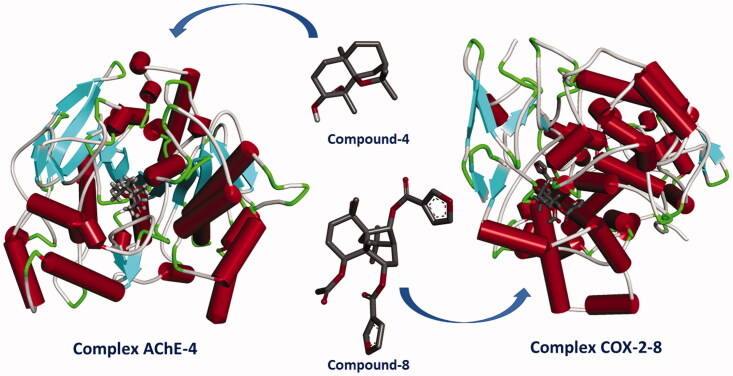

## 1. Introduction

Alzheimer's disease (AD) is a dementia of unknown aetiology that affects adults, usually over 65 years of age^1^. It is a chronic neurodegenerative disease, which causes a progressive and irreversible decrease in cognitive functions, the onset of the disease being characterised by memory loss. AD is often manifested histologically by synaptic and neuronal loss, intracellular neurofibrillary tangles (NFT), and deposition of β-amyloid (Aβ) plaques in human brain^2^. There are several therapeutic targets that can be influenced in order to treat the disease improving the symptoms related to AD or modify its course^3^. The most widely used therapeutic strategy is cholinergic symptomatic therapy, which is based on balance and regulation of neurotransmitters such as acetylcholine.

Acetylcholinesterase (AChE) and butyrylcholinesterase (BChE) are two types of cholinesterase, which catalyse the hydrolysis of choline-based esters to terminate cholinergic signal transmission. These two enzymes differ significantly in substrate specificity, enzyme kinetics, expression and activity in different brain regions and complexity of gene regulation^4^. Both AChE and BChE are therapeutic targets to improve cholinergic dysfunction associated with cognitive and behavioural abnormalities during the development of AD. In the early stages of this disease, the level of AChE increases at a much higher rate than that of BChE in the brain^5^, hence the importance of the use of AChE inhibitors in patients who shown early clinical features of this disease. Currently, inhibitors of AChE are the main strategy for the development of drugs against AD, since by inhibiting this enzyme, the levels of acetylcholine in cholinergic neurons may increase and cholinergic function could improve.

Several decades have passed since the detection of inflammatory mediators in sections obtained from post-mortem brains tissues of AD patients. This findings led to the proposal that neuroinflammatory events could promote AD progression^6^. Recently, in vivo studies using single photon emission (PET) and positron emission computed tomography (SPECT) of AD transgenic mice and AD patients further confirmed that neuroinflammation precedes the outbreak of AD. Therefore, the presence of innate immune cells and the synthesis of inflammatory biomarkers are linked to the progression and severity of the disease^7^. It is suggested that microglia can be activated by Aβ peptides, releasing cytokines and neurotoxins, as if they were responding to a pathogen, which cause neuronal damage, thus mediating neurodegeneration. COX enzymes converts arachidonic acid to inflammatory prostaglandins and thromboxane. These reactions are driven by two isoforms of this enzyme involved in prostaglandin biosynthesis, COX-1 and COX-2. COX-1 is known as a household enzyme and is constitutively expressed in all tissues, while COX-2 is constitutively expressed in the kidneys, brain, and ovaries. COX-2 is expressed during inflammatory conditions by pro-inflammatory molecules such as IL-1, TNF-α, LPS and is practically absent in healthy individuals^8^. In this sense, early epidemiological data suggest that long-term treatment with nonsteroidal anti-inflammatory drugs (NSAIDs) reduces the risk of AD due to the inhibition of the cyclooxygenase 2 (COX-2) up-regulation promoted by Aβ in glial cells^9^. The stimulation of COX-2 in the microglia promote the synthesis of IL-1β, which once secreted stimulate COX-2 in neuron cells leading to the activation PI3-K/AKT and PKA/CREB pathways. In consequence, this cycle is complete when both kinases phosphorylate NF-kB enabling the transactivation of BACE-1, responsible for APP cleavage to produce more Aβ. These events connect the altered expression of COX-2 in the hippocampus with the severity of dementia.

COX-2 inhibitors are widely prescribed anti-inflammatory agents, while COX-1 inhibitors have been associated with several unwanted side effects^10^. Therefore, the development of compounds that exclusively inhibit COX-2 is an important goal to reduce adverse side effects during non-steroidal anti-inflammatory treatment, thus enhancing therapeutic benefits.

Both isoenzymes have similar structures and catalytic activities, the amino acid sequences for the catalytic and binding sites of the substrate are almost identical. So, the main difference between these enzymes lies in that COX-2 has valine instead of isoleucine at positions 434 and 523^11^ Valine is smaller than isoleucine because of a methyl group. These substitutions result in a larger and more flexible substrate channel and a secondary internal pocket outside the COX-2 inhibitor binding site, which is not seen in COX-1.

The Celastraceae family is widespread in tropical and subtropical regions of the world, including North Africa, Central and South America, and Central and East Asia^12^. The plants of this family have a long tradition of use in folk medicine and agriculture. The crude extracts of plants of this family have shown an extraordinary variety of pharmacological properties, among which we can mention their use in digestive disorders, fever, arthritis, bacterial infections and cancer. In addition, these plants are used as stimulants, appetite suppressants and insect repellants^13^. The bulk of bioactive constituents of Celastraceae representative are terpenes, being the sesquiterpene dihydro-β-agarofuran the most characteristic^14^, and the quinone methide triterpenoids (called celastroids). Both class of molecules are recognised as chemotaxonomic markers in this family. Dihydro-β-agarofuran sequiterpenoids show a high degree of oxidation and can form polyesters and pyridine-sesquiterpenes alkaloids. These biosynthetic derivatives have a wide spectrum of biological activities such as immunosuppressants, chemopreventives, anti-HIV, anti-insect agents and to circumvent multidrug resistance (MDR) in cancer cells^15^.

Considering the above mentioned information, in the present work we have evaluated the inhibitory activity of synthetic and natural dihydro-β-agarofurans focussed in acetylcholinesterase and COX enzymes. Our findings suggested that dihydro-β-agarofuran sequiterpenes has significant inhibitory activity, which would allow further design or modification of these molecules in order to improve their potential use in the treatment of AD.

## 2. Results and discussion

### 2.1. Synthetic dihydro-β-agarofurans

**Figure 1.**
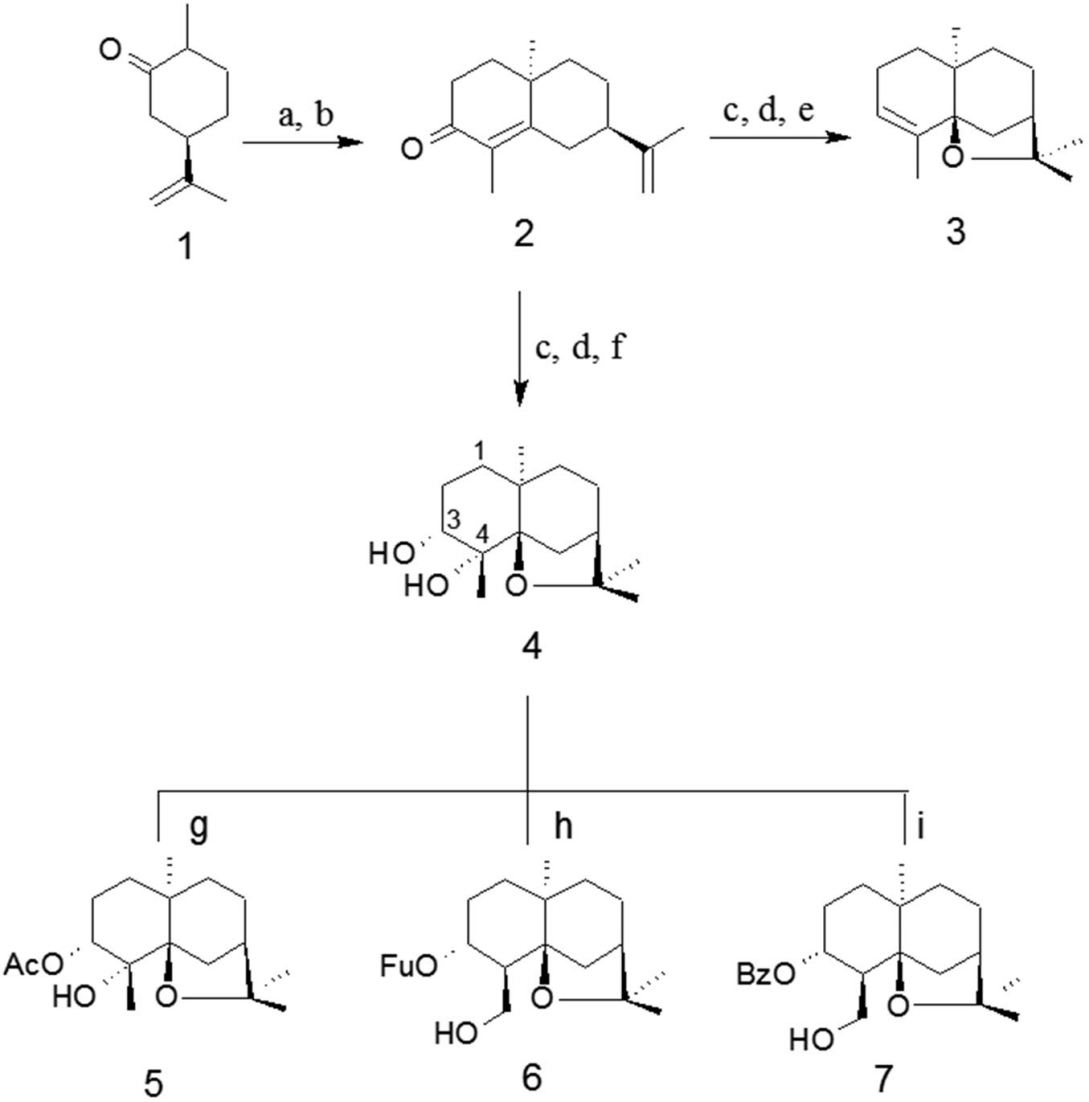
Synthesis scheme of synthetic Dihydro-β-agarofuranoids. a: EVK/KOH; b: Reflux/KOH; c: MCPBA/CH_2_Cl_2_ r.t.; d: LiAlH_4_/(C_2_H_5_)_2_O, 0 °C; e: H_2_SO_4_/Toluene; f: MCPBA/CH_2_Cl_2_ r.t.; g: CH_3_COCl/Py r.t.; h: C_5_H_3_ClO_2_/DMAP/Py 70 °C; i: C_7_H_5_ClO/DMAP/Py 70 °C.

The synthesis of five compounds with dihydro-β-agarofuran skeleton by Robinson´s annulation reaction from (±)-dihydrocarvone and ethyl vinyl ketone was development as outlined in Figure 1 and the structures were identified by their melting point, FTIR, ^1^H NMR, ^13 ^C NMR, and HRMS spectral data. Stereochemistry was unambiguously confirmed with the help of cross-peak intensities observed in nuclear overhauser effect (NOE) and 2 D NOESY (nuclear overhauser effect spectroscopy) spectrum.

Following a published procedure^16^, an epimeric mixture of 10-epiedusmane-4-ene-3,11-diol **4** was prepared in four steps starting from (+)-dihydrocarvone. Treatment with H_2_SO_4_, afforded α-agarofuran **3**. ^1^H NMR spectrum of compound **3** show four singlet signals at δ 1.26, 1.13, 1.14 and 0.81 ppm corresponding at the methyl groups in position C-12, C-13, C-14 and C-15. Distortionless enhancement by polarisation transfer (DEPT) spectra in combination with HSQC (Heteronuclear single quantum coherence) data indicate that compound **3** has a skeleton with 15 carbons, including four methyl carbons at δ C 29.84 ppm (C-12), 24.17 ppm (C-13), 18.72 ppm (C-14), and 21.51 ppm (C-15), five methylene carbons at δC 32.04 ppm (C-1), 22.32 ppm (C-2), 34.05 ppm (C-6), 22.45 ppm (C-8) and 32.53 ppm (C-9), two methine carbons at δ C 73.53 ppm (C-3) and 43.68 ppm (C-7), and four quaternary carbons at δ C 76.29 ppm (C-4), 88.50 ppm (C-5), 38.33 ppm (C-10), and 82.44 ppm (C-11). The carbons C-5 and C-11 appeared displaced to the low field due to the influence of the oxygen of the epoxide ring (δ 80.49 and 84.53 ppm).

**Figure 2.**
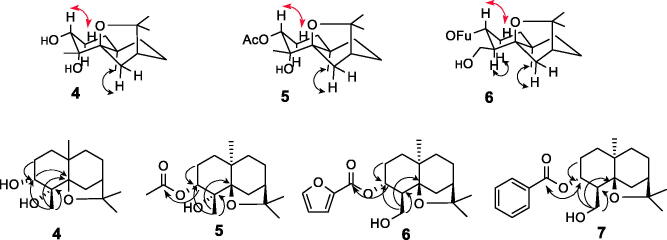
Key nuclear Overhauser effects (NOEs) of compounds **4 **−** 6**, and HMBC correlation of compounds **4**–**7**. Red arrows indicate the NOE interactions of the protons indicated in the compounds **4**–**6** and black arrows indicate the HMBC correlations of the compounds **4–7.**

Spectral data of compound **4** indicates that this has an OH group at the C-4 position, which was confirmed by a singlet at δ 1.32 ppm in the ^1^H NMR spectrum and a quaternary carbon atom at δ 76.29 ppm in the ^13 ^C NMR. The H-3 coupling, attached to the C-3 carbon, is seen as two double signals with an average shift of 3.58 (*J*= 5.1; 10.1 Hz) accounting for an axial proton at this centre. The methyl group at C-4 is in the equatorial position because it showed strong NOEs with H-3 and with the equatorial H-6, while the axial H-6 displayed NOE with the methyl group at C-10 (Figure 2). The ^13 ^C NMR and DEPT (Distortionless Enhancement by Polarisation Transfer) spectra in combination with HSQC data indicate that compound **4** has an agarofuran skeleton with 15 carbons, including four methyl carbons at δ C 22.79 ppm (C-12), 21.56 ppm (C-13), 23.79 ppm (C-14), and 30.50 ppm (C-15), five methylene carbons at δ C 24.35 ppm (C-1), 32.39 ppm (C-2), 35.41 ppm (C-6), 26.59 ppm (C-8) and 37.55 ppm (C-9), two methine carbons at δ C 73.53 ppm (C-3) and 43.68 ppm (C-7), and four quaternary carbons at δ C 76.29 ppm (C-4), 88.50 ppm (C-5), 38.33 ppm (C-10), and 82.44 ppm (C-11). Esterification of compound **4** with acetic anhydride yields compound **5**. ^1^H NMR of this compounds shows an additional methyl at 2.07 ppm, corresponding to methyl acetate. The position of the acetate group was further confirmed by the HMBC (heteronuclear multiple bond correlation) correlations of H-3 (δ 4.86 ppm) with carbonyl at δ170.50 ppm.

Compound **6** was obtained by esterification of **4** with furoyl chloride. The ^1^H NMR spectrum of compound **6** show the presence of three singlet signals in high field that were assigned to the methyl groups in C-12, C-13, and C-15. In addition, it is observed in this compound an AB system between at 4.01–3.98 (*J*= 6.5 Hz) and 3.65–3.63 (*J*= 8.6 Hz), that is not present in the precursor. Further comparison of the ^1^H and ^13 ^C NMR spectroscopic data of compound **4** to those of compound **6** reveals that compound **6** contains one less methyl group and one more methylene bearing a hydroxyl group. This is further confirmed by the HMBC correlations of H-14 (δ H 3.99) with C-4 (δ C 47.57 ppm), C-3 (δ C 76.71 ppm) and C-5 (δ C 89.91 ppm). Similarly compound **7** was prepared by esterification of **4** with benzoyl chloride. The ^1^H NMR spectrum of compound **7** show the presence of three singlet signals in high field that were assigned to the methyl groups in C-12, C-13, and C-15. In addition, it is observed in this compound an AB system between at δ 3.91–3.88 (*J*= 8.0 Hz) and 3.55 − 3.52 (*J*= 10.6 Hz).

These signals are possibly due to an intramolecular rearrangement that facilitated the migration of the hydroxyl group that was present at the C-3 position to the C-14 position. This migration would be facilitated by the use of 4-dimethylamino pyridine (DMAP) as catalyst.

### 2.2. Natural dihydro-β-agarofurans

**Figure 3.**
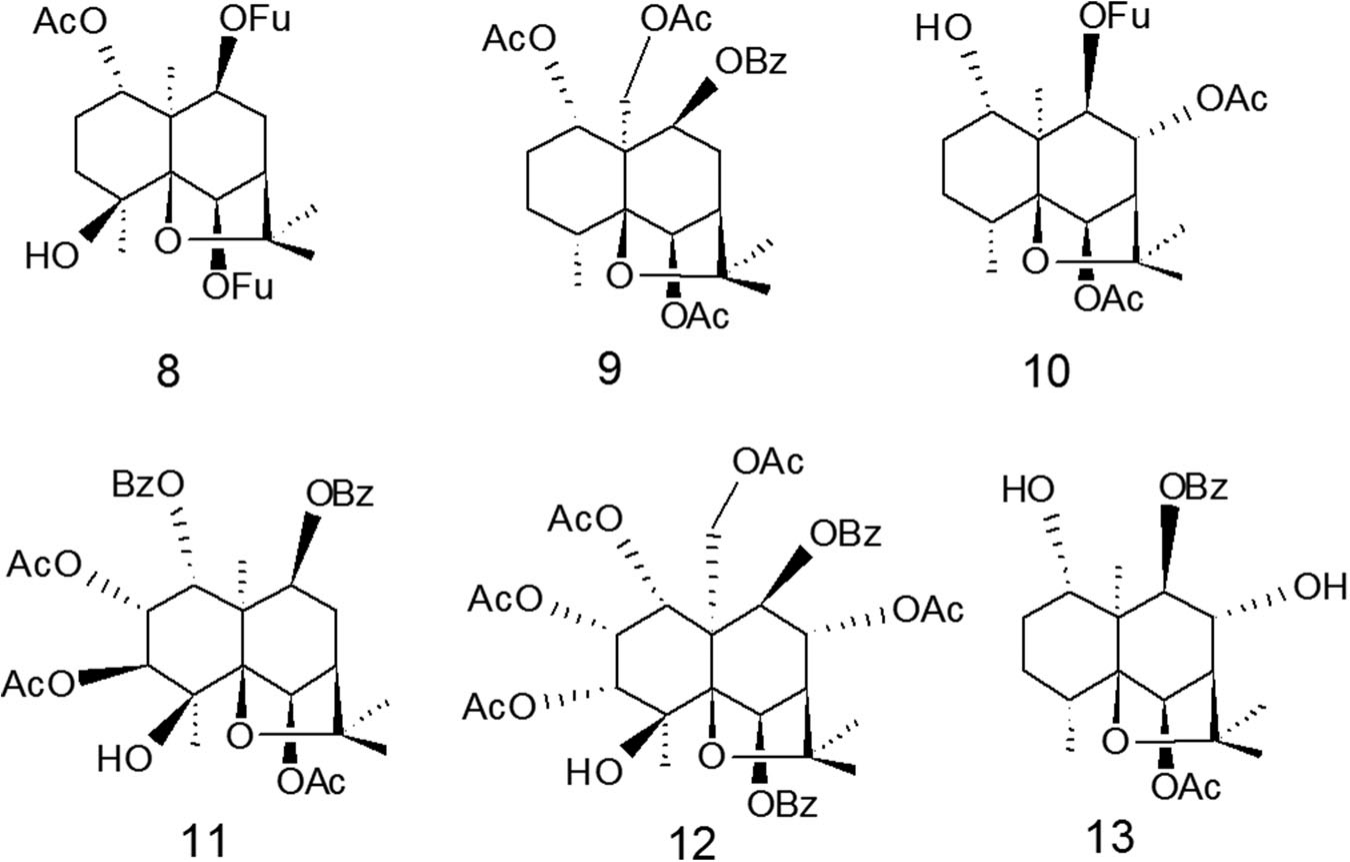
Chemical structure of the natural Dihydro-β-agarofuran evaluated.

The chemical study of the fractions obtained from the methanolic extract of the *Maytenus disticha* and *M. magellanica* seeds by CC and preparative TLC resulted in the isolation of six compounds (Figure 3). Compounds **8**, **10** and **13** were isolated from *M. disticha* and compounds **11**, **12** and **9** were isolated from *M. magellanica*. Their NMR spectra showed signals characteristic of dihydro-β-agarofurans^17–20^. The ^1^H NMR spectrum of compound **8** showed the typical signals of a β- substituted furan ring (δ 6.72; 7.40; 8.01 ppm) in C-9 and the change of field down to δ 5.53 of H-6 indicates the relative position of a second furan group ester in C-6. The double doublet at δ 5.27 (1H, dd, *J*= 4.0, 12 Hz) was assigned to H-1 because in this class of compound H-1 had generally axial stereochemistry. Furthermore, typical signals were present for eudesmane with an angular methyl group (H-15) at 1.34 ppm (s), a secondary methyl group (H-14) at 1.40 ppm (s), and singlets at 1.51 ppm (H-12) and 1.53 ppm (H-13), the signals at 2.08 ppm (s) corresponded to protons of acetate group. Together with the ^13 ^C NMR spectrum, the presence of the eudesmane system with oxygen functions at C-1, C-4, C-5, C-6, and C-9 was established. Compound **10** also exhibits characteristic signals for a furan ring at position C-9 (δ 8.08, 6.75 and 73.45 ppm) and the signals at δ 2.08 (s) and δ 2.38 ppm corresponded to protons of acetate group in C-8 and C-6 respectively.

Compound **12** has the highest number of substitutions: five groups of acetates (δ 2.16, 2.11, 2.08, 1.97, 1.92 ppm), a hydroxyl group at position C-4 (1H, s, δ 3.93 ppm) and group benzoyl in the C-6 position (5H, m, δ 8.12–8.03 ppm). The proton at position C-9 is visualised as a doublet (*J*= 4.0 Hz) at δ 5.17 ppm, while the proton at C-6 is seen as a doublet at δ 4.83 (*J*= 2.1 Hz). The ^1^H NMR spectrum to compounds **12** and **9** showed different signals to the rest of the compounds at δ 4.72–4.68 (1H, m) and 4.68 (1H, d, *J*= 12.0 Hz) respectively, this is due to the presence of the acetyl group in C-15. Compound **7** too showed a double doublet (*J*= 12.5 and 5.0 Hz) at δ 5.55, this signal was assigned to the proton H-1 in the axial position, the proton H-9 is seen as a doublet (*J*= 7.5 Hz) and the proton H-6 is seen at δ 5.95 ppm (br s).

The ^1^H NMR spectrum to compounds **11** and **13** confirmed the presence of benzoate groups between δ 8.16–7.89 and δ 7.61–7.37 ppm to **11** and δ 8.10 ppm (dd, *J*= 1.0; *J*= 7.1 Hz) to **13**. Methyl groups for acetates for compounds **11** and **13** appeared as singlets between δ 2.10 and 2.24 ppm depending chemical environment of the molecule.

### 2.3. Inhibition of the activity of the enzyme acetylcholinesterase and COX 1/2 and kinetic study of the inhibition of AChE

All compounds synthesised were evaluated *in vitro* as dual AChE and BChE inhibitors according to the modified Ellman’s method^21^. Galantamine and carvacrol, both inhibitors of AChE, were used as the positive controls. The concentration of the compound required for 50% enzyme inhibition (IC_50_) was calculated by means of regression analysis. The results of this assay are listed in Table 1. IC_50_ values are expressed in µM as means ± SEM and were compared by using ANOVA analysis. A *p* values of less than 0.05 was considered significant.

**Table 1.** IC_50_ values and Selectivity index (SI) of *in vitro* COX-1/COX-2 and AChE enzymes inhibition assay.

Compounds	AChE IC_50_ (µM)±SE	COX-1 IC_50_ (µM)±SE	COX-2 IC_50_ (µM)±SE	SI(COX-1/COX-2)
**3**	26.0 ± 0.012	11.36 ± 0.035	0.17 ± 0.002	66.82
**4**	17.0 ± 0.016	14.21 ± 0.035	0.38 ± 0.007	37.39
**5**	43.0 ± 0.015	13.08 ± 0.02	0.40 ± 0.0014	32.7
**6**	32.0 ± 0.006	12.69 ± 0.035	0.13 ± 0.003	97.61
**7**	n.a.	11.98 ± 0.042	0.21 ± 0.008	57.05
**8**	738.0 ± 0.007	10.59 ± 0.02	0.04 ± 0.007	264.75
**9**	695.0 ± 0.001	8.69 ± 0.03	0.07 ± 0.004	124.14
**10**	740.0 ± 0.045	13.74 ± 0.1	0.29 ± 0.0003	47.37
**11**	30.0 ± 0.06	13.08 ± 0.02	0.09 ± 0.005	145.33
**12**	n.a.	14.21 ± 0.2	0.84 ± 0.012	16.92
**13**	500.0 ± 0.03	11.36 ± 0.035	0.11 ± 0.007	103.27
Galantamine	10.0 ± 0.015	–	–	–
Carvacrol	45.0 ± 0.031	–	–	–
Celocoxib	–	13.01 ± 0.1	0.038 ± 0.004	342.36
Diclofenac Na	–	2.6 ± 0.025	0.61 ± 0.012	4.26

*Data are the average of 3 independent runs ± SD*.

As it can be observed in Table 1, all the evaluated compounds show inhibitory activity of acetylcholinesterase enzyme. The IC_50_ values were from 17.0 ± 0.016 to 740.0 ± 0.045 µM. The results obtained are satisfactory in relation to the reference compounds used (galantamine and carvacrol), and when compared with other terpenoids of natural origin^22^. None of the compounds evaluated showed significant inhibitory effects on the BChE enzyme (IC_50_ >500 µm, data not show). Therefore, these compounds can serve as selective inhibitors for AChE, thus avoiding unexpected effects on peripheral tissues due to BChE inhibition.

Compound **4** was the most potent anti-AChE agent with an IC_50_ value of 17.0 ± 0.016 µM to AChE, which makes it significantly better than natural agarofurans isolated from previously reported^16^^,^^23^. Inhibitory activity is probably favoured by free hydroxyl groups at positions C-3 and C-4, which favour the formation of hydrogen bonds with the residues of the enzyme.

**Figure 4.**
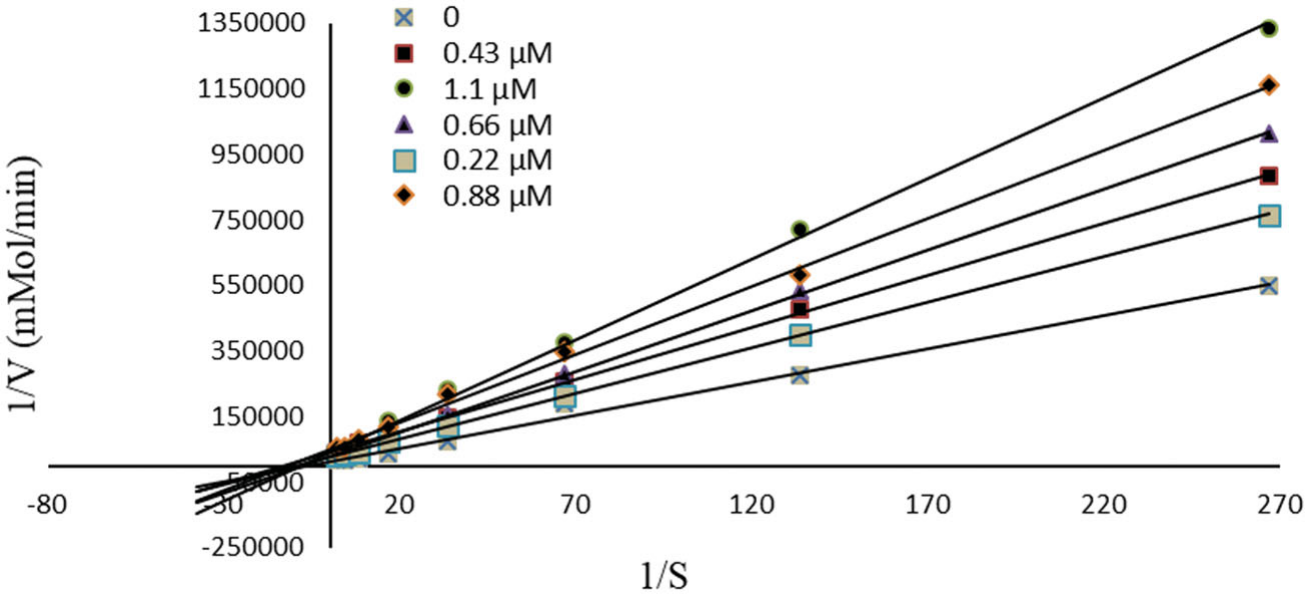
Kinetic study on the inhibition mechanism of AChE by compound **4**.

Based on the obtained results in the cholinesterase inhibition assay, and with the aim of assessing the kinetic mode of AChE inhibition of the target compounds, the most active compound **4** was evaluated. The rate of enzyme activity was measured at eight different concentrations of substrate acetylthiocholine iodide (ATC). After plotting the reciprocal of enzyme velocity (l/v) versus the reciprocal of substrate concentration (l/S), the established Lineweaver-Burk reciprocal plots (Figure 4) showed that the Vmax decreases in the presence of the inhibitor, while the intercepts remain unchanged, the value of Km = 0.11 mM being at increasing concentrations of the inhibitor for Tc-AChE. These kinetic parameters would indicate that **4** is a non-competitive inhibitor.

Recognising the action mode of enzyme inhibitors is very important; however, much of the drug discovery process only focuses instead upon stablishing whether one inhibitor is more powerful than another. Reversible inhibition comprises of the ensuing subtypes: competitive, uncompetitive, non-competitive or mixed-type. Such inhibitions can be determined using Lineweaver–Burk plots. When a molecule binds to the catalytic active site (CAS), the type of inhibition of the enzyme is classified as competitive; by contrast, when a molecule interacts with the peripheral anionic site (PAS), the enzymatic inhibition is called non-competitive^24^. Furthermore, once the active molecule acts on both CAS and PAS, the enzymatic inhibition is designated as mixed inhibition. Considering the above mentioned, our kinetics study for the compound **4** suggest an interaction with the PAS reflexed by a lower Vmax value and unchanged Km.

In a previous work carried out by Alarcón et al.,^25^ sesquiterpene esters with a dihydro-β-agarofuran skeleton were isolated from Chilean Celastraceae and it was found that most of the compounds had inhibitory activity on AChE. The IC_50_ values for said compounds were found in the range of 0.046 to 0.738 mM. When comparing the results, it can be seen that the synthesised dihydro- β -agarofurans have a better AChE inhibitory activity than the compounds isolated from plants. These results show that there would be a possible relationship between the size of the substituents surrounding decalin and its inhibitory activity. Synthetic compounds with less bulky groups would have better AChE enzyme inhibitory activity.

The synthesised compounds were evaluated as COX-1 and COX-2 inhibitors. The IC_50_ values of the compounds against COX-1 ranged from 8.69 ± 0.03 to 14.21 ± 0.2 µM, while the IC_50_ values obtained for COX-2 ranged from 0.04 ± 0.007 to 0.84 ± 0.012 µM as shown in Table 1. The compounds showed prominent inhibition constants towards both COX isoforms comparable to those of the standard drugs used, diclofenac sodium and celecoxib. It can be seen that dihydro-β-agarofurans show more potent and selective inhibitory effects on the COX-2 isoform. Interestingly, the best activities come from the naturally occurring compounds **8–13**, while the synthetic compounds show a diminished effect in comparison.

It is worthy to note that, structural variations appeared to affect both the potency and the selectivity of these derivatives against the enzymes tested. This is a specially sought trait for the development of selective anti-inflammatory agents to avoid undesirable side effects.

Regarding the results of the COX-2 enzyme inhibition assay, compound **8** was the most potent, showing lower IC_50_ value 0.04 ± 0.007 µM and SI 264.75, compared to the known selective COX-2 inhibitor Celecoxib (IC_50_; 0.049 µM and SI; 308.16).

The increase in the initial expression of COX-2 and in the enzymatic activity promotes the appearance of neuronal lesions in various models of neurological disease. It is an interesting approach to alter the microglial inflammatory response through COX-2 inhibition^26^. Pharmacological inhibition of COX-2 is neuroprotective in rodent models of stroke^27^. Inhibition of COX-2 enzyme activity with non-steroidal anti-inflammatory drugs has been shown to reduce inflammation in murine transgenic models of AD, as well as the use of COX-2 inhibitors to decrease the risk of developing AD in populations. healthy ageing^28^. COX-mediated neuronal injury is presumed to be due to downstream effects of one or more prostaglandin products including PGE2, PGD2, PGF2α, PGI2 and TXA2 that affect cellular changes through activation of specific prostaglandin receptor subtypes. The suppression of these proteins has been linked to improved memory in transgenic AD mouse models^29^.

### 2.4. Docking Studies on AChE and COX-1/2

To gain insight into the binding interactions of compounds with enzymes, molecular coupling studies were carried out. The data in Table 2 show the free binding energies (−9.1 to −4.2 kcal/mol) of each compound to the enzyme AChE. The most active compound **4** formed three H bonds with AChE. The hydroxyl group at C-3 forms two hydrogen bonds with the residue Arg 289 (1.9 and 1.73 Å) and the hydroxyl group at C-4 forms a hydrogen bond with the residue of Ser 286 (2.14 Å). In addition, the methyl groups at the C-12 and C-13 positions interact through a π-sigma bond with residue Trp 279; The C-1 and C-12 carbons form π -alkyl bonds with the amino acids Tyr 334 and Tyr 70. Finally, several Van der Waals interactions were observed with Gly 335, Ile 287, Leu 282, Phe 331, Phe 288 and Phe 290 The amino acids Trp 279, Tyr 334, and Tyr 70 belong to the peripheral anionic enzyme site.

**Table 2.** Binding energy of compounds evaluated (kcal mol^−1^).

Compounds	AChE	COX-1	COX-2
**3**	−8.2	−6.9	−6.8
**4**	−9.1	−4.4	−5.9
**5**	−6.1	−5.9	−5.0
**6**	−6.3	−6.0	−7.1
**7**	–	−6.3	−6.3
**8**	−4.5	−7.2	−7.9
**9**	−4.6	−7.3	−7.7
**10**	−4.5	−5.0	6.0
**11**	−6.7	−5.4	−7.4
**12**	–	−5.0	−3.3
**13**	−4.2	−7.1	−7.3
Galantamine	−10.8	–	–
Carvacrol	−6.4	–	–
Celocoxib	–	−5.3	−7.9
Diclofenac Na	–	−7.8	−6.3

The rest of the compounds evaluated interact in a similar way with some of the amino acids that are part of the PAS. In addition, all of them, with the exception of compound **11**, interact through π bonds with amino acids located in the gorge centre of the enzyme such as Phe 330, Trp 279, Phe 290 and Phe 331. These interactions are important because these amino acid residues connect the peripheral site and the anionic subsite in the active site of the enzyme. When inhibitor entering the enzymés active site, possibilities that substrate can reach the bottom of the gorge are limited^30^. This way, dihydro-β-agarofurans would obstruct the entrance of the substrate to the active site. The PAS is responsible for non-cholinergic functions of AChE and plays a role in the deposition of amyloid plaque. Therefore, molecules that bind to PAS site could, plays an important role slowing down the progression of AD^31^.

AChE has been recognised as a target for a number of toxins and promising drugs such as huperzine^32^ and derivates, propidium iodide^33^, gallamine triethiodide^34^ among others. In order to maintain a stable binding of these inhibitors with the enzyme, many cooperative interactions are established such as hydrogen bonds, hydrophobic contacts, π-π (aromatic) interactions, and hydrophilic-hydrophobic interactions^35^. The chemical structure of the evaluated compounds allows many of these interactions to be formed. For example, the free hydroxyl groups, the epoxide group and the carbonyl groups can form hydrogen bonds; the methyl groups generate nonpolar interactions and it is also feasible to form π-type interactions with the aromatic amino acids that are part of the PAS portion of the enzyme^36^.

**Figure 5.**
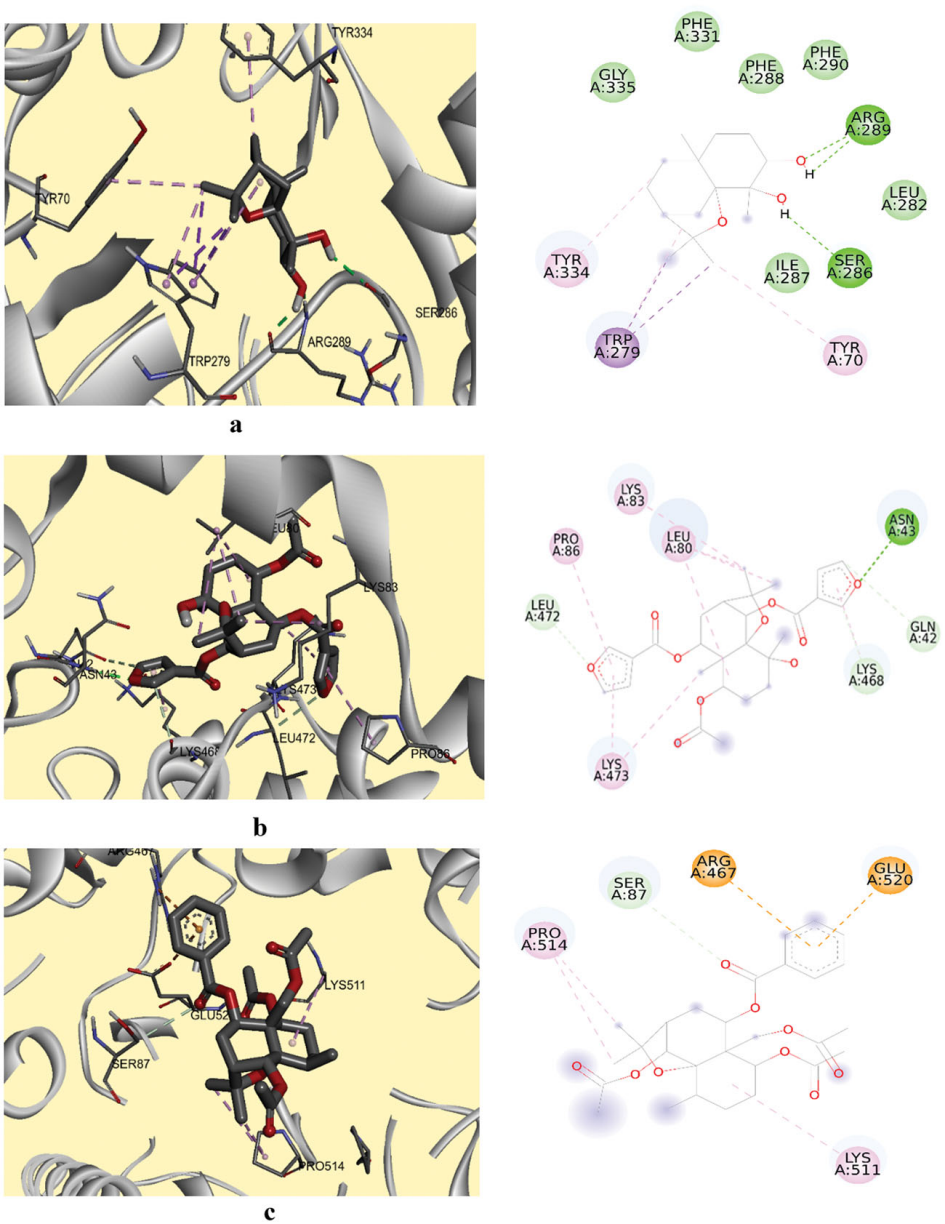
2D and 3D schematic diagram of the docking model of a) compound **4** with AChE, b) **9** with COX-1 c) **8** with COX-2.

In our study, the best inhibition of the COX-1 enzyme was given by compound **9**. Molecular docking for this ligand-enzyme complex shows that one non-conventional hydrogen bond is formed between the benzoyl group at position C-9 with the Ser 89 residue, π-cation and anion interactions with Arg 467 and Glu 520 residues and several nonpolar interactions of the π and π alkyl type were generated with the residues of Pro 514 and Lys 511 (Figure 5).

The data in Table 2 show the free binding energies (−7.9 to −3.3 kcal/mol) of the compounds to the enzyme COX-2. The most active compound **8** formed one conventional hydrogen bond between Asn 43 residue and the furoyl group of C-6 position, three non-conventional hydrogen bonds between the furoyl groups at position C-6 and C-9 and the residues of Gln 42, Lys 468 and Leu 472 and several non-polar interactions of the type π and π alkyl with residues Lys 473, Leu 80, Pro 86 and Lys 83.

### 2.5. Displacement of propidium iodide from the peripheral anionic site

The fluorescent assay used to evaluate competitive propidium iodide displacement from the PAS of AChE is commonly used as primary screening method of AChE pro-aggregation activity inhibitors. Propidium iodide is a selective ligand for the PAS of AChE responsible for Aβ binding. It exhibits a fluorescence increase upon binding to AChE. A decrease in propidium iodide fluorescence in the presence of test compounds suggests that they are able to displace propidium, consequently bind to the PAS of AChE^37^.

Compounds **3**, **4 **y **11** were examined for their ability to bind to the PAS of AChE and displace propidium iodide. The three compounds evaluated decreased fluorescence intensity by 12.1% and 13.9% at 290 µM, and by 13.4 and 17.6% at 340 µM (Table 2). Our data suggest that the conjugates are able to bind to the PAS of AChE and therefore might be able to inhibit the aggregation of amyloid peptides induced by AChE. The above is supported by studies demonstrating that ligands binding selectively to the PAS, such as propidium iodide, are capable of blocking Aβ aggregation. Therefore, the development of drugs blocking site PAS in AChE and prevent interactions with β-amyloid peptide (and thus decreasing its AChE-induced aggregation) is a promising approach for anti-amyloid AD treatment^38^.

### 2.6. Prediction of absorption, distribution, metabolism, excretion and toxicity (ADMET) and related properties

The pharmacokinetic profile, that is, the absorption, distribution, metabolism and excretion (ADME) of the compounds was predicted with the help of SWISSADME (http://www.swissadme.ch/index.php). The application was used for in silico prediction of drug similarity of all compounds based on various molecular descriptors and the results are presented in Table 3. Druglikeness plays an effective role in drug design for a new drug (molecules studied) and is generally based on five basic rules (RO5) defined by Lipinski^39^. Lipinski's RO5 is a general rule of thumb for determining drug similarity and determining whether an inhibitor with specific biological and pharmacological characteristics can be an orally active drug in the human body. Furthermore, from the SwissADME website it was possible to obtain properties such as log S (Ali: aqueous solubility), molar refractivity values and inhibitory (+) and non-inhibitory (-) properties) of CYP450 enzymes such as CYP1A2, CYP2C19, CYP2C9, CYP2D6 and CYP3A4.

**Table 3.** Inhibition of AChE and displacement of propidium iodide from the PAS.

Compound	IC_50_ µM	% Displacement of propidium iodide	
		290 µM	340 µM
**3**	26.0 ± 0.012	12.2 ± 0.2	15.8 ± 0.5
**4**	17.0 ± 0.016	13.9 ± 0.3	17.6 ± 0.5
**11**	30.0 ± 0.06	12.1 ± 0.7	13.4 ± 0.2
Donepezyl	0.072 ± 0.007	22.2 ± 0.9	24.1 ± 0.6

*Data are the average of 3 independent runs ± SD*.

Negative BBB values were obtained for natural compounds **8**–**13** and positive values for synthetic compounds **3**–**7**, which coincides with the best AChE inhibition obtained experimentally. The values of human intestinal absorption that is, the use of molecules in the human body (a crucial barrier in the formulation of new drugs), were high in most of the compounds, both synthetic and natural and only the compounds **8**, **11** and **12** showed low absorption. Pan-Assay interference compound (PAINS) index was negative for all the compounds and, as can be seen in Table 3, most of the compounds comply with the five Lipinski rules.

## 3. Experimental

### 3.1. General experimental procedures

NMR spectra, including COSY, NOESY, HMBC, and HSQC experiments, were recorded on a Bruker spectrometer operating at 300 and 400 MHz (^1^H) and 100 MHz (^13 ^C), respectively, using CDCl_3_ and DMSO-d_6_ as solvents. Tetramethylsilane (TMS) was used as an internal standard. IR spectra (KBr pellets, 500–4000 cm^− 1^) were recorded on a NEXUS 670 FT-IR spectrophotometer. The compounds were analysed by to high-resolution mass spectrometry (HPLC‐HRMS/MS) on a maxi G3 quadrupole time‐of‐flight mass spectrometer (Bruker Daltonics, Bremen, Germany) equipped with an ESI source. The MS was connected to a Shimadzu CBM‐20A system with communication module CBM‐20A, diode‐array detector (DAD) SPD‐20A and a Phenomenex Luna C‐18 reversed‐phase column (250 mm × 4.6 mm, 5 µm) (Torrance, CA). The ^1^H RMS analysis was carried out in positive mode (ESI+) with a data acquisition range of 2 scans/s at m/z 50–1500. The eluent flow of 200 µL/min was carried to the ion source. Dry nitrogen (N_2_) was used as nebuliser gas, with a pressure of 2 bar, corresponding to a gas volume flow of 8 L/min. The other ESI conditions: capillary tension of 4500 V, end plate offset of 500 V, drying temperature of 200 °C, collision energy of 6.0 eV. Analytical and preparative TLC was performed on Silica Gel 60 Merck plates, and the spots visualised by spraying with a 10% solution of H_2_SO_4_, followed by heating at 110 °C. All reagents were purchased from either Merck or Sigma Aldrich (St. Louis, MO, USA) and used without further purification.

### 3.2. Synthesis of the dihydro-β-agarofurans 3 and 4

Agarofuran **3** and **4** (60 and 90% yield respectively) were both prepared in five steps from (+)-Dihydrocarvone as described by Alarcón et al ^16^. Compounds **5** to **7** are reported for the first time both their structure and their activity.

### 3.3. Esterification of 4 to the preparation of compounds 5–7

Compound **4** (1.49 mmol) was dissolved in 250 µL of anhydrous pyridine were heated at 70 °C and under stirring with 1.49 mmol of CH_3_COCl and 0.75 mmol of 4-dimethylamino pyridine (DMAP) for 1 h. The resulting mixture was diluted with water, allowed to stand for 30 min, extracted with EtOAc and dried with anhydrous sodium sulphate. Removal of the solvent gave a residue which crystallised from n-hexane in the form of a colourless fine needle of **5** (yield 99%). Compounds **6** and **7** were prepared in the same manner using furoyl chloride (C_5_H_3_ClO_2_) and benzoyl chloride (C_7_H_5_ClO) respectively (95% and 97% yield respectively). The final purification of all the products for analysis was carried out by recrystallization.

### 3.4. Extraction and separation from seeds of maytenus disticha and Maytenus magellanica

From 300 g of *M. disticha* seeds and 200 g of *M. magellanica* were macerated separately in MeOH at room temperature for 48 h. After this time the seeds are filtered and the solvent evaporates. The resulting extract was fractionated with CHCl_3_, EtOAc, and H_2_O. Compounds **8**, **10** and **13** were obtained from the CHCl_3_ fraction of *M. disticha* seeds, while compounds **9**, **11** and **12** were obtained from *M. magellanica*.

### 3.5. Spectroscopic data

*Compound* ***3****; α-agarofuran:* [α]_D_^15^= +36.8 (c = 1.0, CHCl_3_); Yield: 60%, yellow oil; IR (KBr, cm^−1^): 1653, 1389, 1370, 1326, 1307, 1285, 1250, 1136, 1099, 1046, 965, 950, 935, 856, 825, 805. ^1^H NMR (300 MHz, CDCl_3_, δ in ppm) δ = 5.45 (1H, s, H-3), 1.26 (3H, s, H-12), 1.13 (3H, s, H-13), 1,14, (3H, s, H-14), 0.81 (3H, s, H-15). ^13 ^C NMR (100 MHz, in ppm): δ = 132.15 (C-4), 127.03 (C-3), 84.53 (C-5), 80.49 (C-11), 43.96 (C-7), 36.61 (C-10), 34.05 (C-6), 32.53 (C-9), 32.04 (C-1), 29.84 (C-12), 24.17 (C-13), 22.45 (C-8), 22.32 (C-2), 21.51 (C-15), 18.72 (C-14). HRMS (ESI, *m/z*): calculated for C_15_H_24_O [M + H] ^+^ 221.1905 found 221.1902.

*Compound* ***4****; 3α,4α-dihydroxydihydro-β-agarofuran:* [α]_D_^20^ −64.9 (c = 1.0, CHCl_3_). Yield: 90%, White solid; mp:109–110 °C; IR (KBr, cm^−1^): 3518, 3380, 2996, 2924, 2864, 1458, 1378, 1088, 1026, 1010, 928, 886; ^1^H NMR (300 MHz, CDCl_3_, δ in ppm) δ = 3.58 (dd, 1H, *J*= 5.1, 10.14 Hz), 2,47–2,19 (1H, dd, *J*= 4.6, 12.4 Hz, H-2), 1.82 (1H, d, *J* = 11.0), 2.15 (1H, s, H-7), 1.31 (3H, s, H-12), 1.28 (3H, s, H-13), 1.23 (3H, s, H-14), 1.16 (3H, s, H-15). ^13 ^C NMR (100 MHz, in ppm): δ = 88.50 (C-5), 82.44 (C-11), 76.29 (C-4), 73.53 (C-3), 43.68 (C-7), 38.33 (C-10), 37.55 (C-9), 35.41(C-6), 32.39 (C-2), 30.50 (C-15), 26.59 (C-8), 24.35 (C-1), 23.79 (C-14), 22.79 (C-12), 21.56 (C-13). HRMS (ESI, *m/z*): calculated for C_15_H_26_O_3_ [M + H] ^+^ 255.1960 found 255.1954

*Compound* ***5****; 3α-acetoxy-4α-hydroxydhydro-β-agarofuran:* [α]_D_^20^ −51.7 (c = 1.0, CHCl_3_). Yield: 99%, White solid; mp:112–113 °C; IR (KBr, cm^−1^): 3514, 2916, 1739, 1449, 1376, 1261, 1227, 1135, 1095, 1009, 954, 870, 796. ^1^H NMR (400 MHz, CDCl_3_, δ in ppm) δ = 4.86, (dd, 1H, *J*= 4.9, 11.5 Hz, H-3), 2.14 (d, 1-H, *J*= 4.9 Hz, H-6), 2.23–2.17 (m, 1H, H-7), 2.07 (3H, s, 3-OAc), 1.13 (3H, s, H-15), 1.16 (1H, s, H14), 1.25 (3H, s, H-11), 1.305 (3H, s, H-12). ^13 ^C NMR (100 MHz, in ppm): δ = 87.96 (C-5), 82.16 (C-11), 76.86 (C-3), 75.70 (C-4), 43.41 (C-7), 38.17 (C-10), 37.25 (C-9), 35.05 (C-1), 32.08 (C-6), 29.71 (C-12), 24.13 (C-13), 23.50 (C-2), 22.79 (C-8), 21.45 (C-14), 20.84 (C-15). HRMS (ESI, *m/z*): calculated for C_17_H_28_O_4_ [M + H] ^+^ 297.2066 found 297.2062.

*Compound* ***6****; 3α-furoyloxy-15-hydroxydihydro-β-agarofuran:* [α]_D_^20^ −104 (c = 1.0, CHCl_3_). Yield: 95%, light yellow solid; mp: 108–109 °C. IR (KBr, cm^−1^): 3540, 2915, 2854, 1696, 1564, 1397, 1186, 1013, 928, 873, 770, 678, 594. ^1^H NMR (300 MHz, CDCl_3_, δ in ppm) δ = 7.7–6.65 (3H, 3-OFu), 5.21–5.17 (1H, m, H-3), 3.99 (1H, d, *J*= 9.0, H-14), 1.83–1.91 (1H, m, H-7), 1.80–1.79 (1H, m, H-2), 1.44 (3H, s, H-12), 1.43 (3H, s, H-13), 1.40 (3H, s, H-15) ^13 ^C NMR (100 MHz, in ppm): δ = 89.91 (C-5), 84.2 (C-11), 76.71 (C-3), 76.01 (C-14), 47.57 (C-4), 43.2 (C-7), 42.07 (C-10), 37.57 (C-6), 32.45 (C-1), 32.23 (C-9), 32.08 (C-12), 30.04 (C-13), 25.29 (C-2), 24.46 (C-8), 24.03 (C-15). HRMS (ESI, *m/z*): calculated for C_20_H_28_O_5_ [M + H] ^+^ 349.2015 found 349.2025.

*Compound* ***7****; 3α-benzyloxy-15-hydroxydihydro-β-agarofuran:* [α]_D_^20^ −13.3 (c = 1.0, CHCl_3_). Yield: 97%, White solid; mp: 117–118 °C. IR (KBr, cm^−1^): 3509, 2907, 1692, 1450, 1281, 1120, 1096, 1012, 975, 928, 884, 710, 587. ^1^H NMR. (300 MHz, CDCl_3_, δ in ppm) δ = 8.07–7.28 (5H, 3-OBz), 5.2–5.3 (1H, m, H-3), 3.91–3.88 (1H, d, *J*= 9.0, H-14), 3.55–3.52 (1H, m, OH), 2.21–1.92 (1H, m, H-4), 1.79–1.78 (1H, m, H-7), 1.25 (3H, s, H-15), 1.27 (3H, s, H12), 1.28 (3H, s, H-13). ^13 ^C NMR (100 MHz, in ppm): δ = 89.73 (C-5), 84.73 (C-11), 77.96 (C-3), 47.56 (C-4), 43.96 (C-7), 42.16 (C-10), 39.76 (C-6), 38.57 (C-1), 35.37 (C-9), 32.17 (C-12), 30.17 (C-13), 25.38 (C-2), 22.98 (C-7), 21.98 (C-15). HRMS (ESI, *m/z*): calculated for C_22_H_30_O_4_ [M + H] ^+^ 359.2222 found 359.2215.

*Compound* ***8****; 1α-Acetoxy-6β,9β-difuroyloxy-4β-hydroxydihydro-β-agarofuran:* [α]_D_^25^= +11.20 (c 0.5, CHCl_3_). Yield: 40 mg, white solid, m.p: 208–212; IR (KBr, cm^−1^): 3480, 3140, 1505,872, 1735, 1113, 706. ^1^H NMR (400 MHz, CDCl_3_, δ in ppm) δ = 5.27 (1H, dd, J = 4.0, 12.0 Hz, H-1), 1.20 (1H, m, H-2α), 1.50 (1H, m, H-2β), 1.90 (1H, m, H-3α), 1.70 (1H, m, H-3β), 5.53 (1H, s, H-6), 2.32 (1H, dd, *J*= 3.0, 3.0 Hz, H-7), 2.51 (1H, m, H-8α), 2.21 (1H, d, *J*= 7.0 Hz, H-8β), 4.98 (1H, d, *J*= 7.0 Hz, H-9), 1.51 (3H, s, H-12), 1.53 (3H, s, H-13), 1.40 (3H, s, H- 14), 1.34 (3H, s, H-15). HRMS (ESI, *m/z*): calculated for C_27_H_32_O_10_ [M + H] ^+^ 517.2074 found 517.2072.

*Compound* ***9****; 1α,6β,14-triacetoxy-9β-benzyloxydihydro-β-agarofuran*: Yield: 14 mg, white solid, m.p:149–150; IR (KBr, cm^−1^): 2928, 1732, 1258, 1100, 717, 559. ^1^H NMR (400 MHz, CDCl_3_, δ in ppm) δ = 5.95 (1H, br s, H-6), 5.55 (1H, dd, *J*= 12.5, 5.0 Hz, H-1), 5.39 (1H, d, *J*= 7.5 Hz, H-9), 4.68 (1H, d, *J*= 12.0 Hz, H-15), 4.46 (1H, d, *J*= 12.0 Hz, H-15), 2.6 (1H, d, *J*= 1.0 Hz, H-7), 2.23 (3H, s), 2.09 (3H, s), 1.52 (3H, s), 1.44 (3H, s, H-13), 1.39 (3H, s, H-12), 0.96 (3H, d, *J*= 8.0 Hz, H-15), 8.05; ^13 ^C NMR (100 MHz, in ppm). Δ = 89.5 (s, C-11), 82.4 (s, C-5), 78.1 (d, C-1), 73.4, 69.9, 65.3 (t, C-14), 53.0 (s, C-10), 48.7 (d, C-7), 34.9 (t, C-8), 33.4 (d, C-5), 26.31 (t, C-2), 22.1 (t, C-3), 16.5 (q, C-15). HRMS (ESI, *m/z*): calculated for C_28_H_36_O_5_ [M + H] ^+^ 453.2596 found 453.2583.

*Compound* ***10****; 6β,8α-diacetoxy-9β-furoyloxy-1α-hydroxydihydro-β-agarofuran*: Yield: 20 mg, white solid, mp. 183–185;IR (KBr, cm^−1^): 3140, 1505, 872, 1735, 1100, 705, 506 ^1^H NMR (400 MHz, CDCl_3_, δ in ppm) δ = 6.03 (1H, s, H-6), 5.35 (1H, br s, H- 9), 5.14 (1H, dd, *J* = 4.0,11.0 Hz, H-1), 4.32 (1H, br d, *J* = 3.5 Hz, H- 8), 1.83 (1H, m, H-2α), 1.56 (1H, m, H-2β), 1.41 (1H, m, H-3α), 2.11 (1H, m, H-3β), 2.22 (1H, m, H-4), 2.46 (1H, br s, H-7), 1.42 (3H, s, H-15), 1.38 (3H, s, H-13), 1.31 (3H, s, H-12), 0.99 (3H, d, *J* = 7.0 Hz, H-14), 2.38 (3H, s), 8.07 (1H, dd, *J*= 1.5, 1.0 Hz), 6.75 (1H, dd, *J*= 1.0, 2.0 Hz, OFu), 7.45 (1H, dd, *J*= 1.5, 2.0 Hz); ^13 ^C NMR (100 MHz, in ppm). δ = 81.2, (s, C-11), 79.3 (s, C-5), 76.1 (d, C-9), 74.6 (d, C-6), 70.1 (d, C-1), 54.1 (d, C-7), 48.5 (s, C-10), 33.7 (d, C-4), 30.6 (q, C- 12), 24.0 (t, C-3), 21.4 (t, C-2), 20.8 (q, C-13), 16.8 (q, C-14), 12.2 (q, C-15). HRMS (ESI, *m/z*): calculated for C_24_H_32_O_6_ [M + H] ^+^ 417.2232 found 417.2229.

*Compound* ***11****;2α,3β,6β-triacetoxy-1α,9β-dibenzyloxy-4β-hydroxydihydro-β-agarofuran:* [α] _D_^20^ + 143.4 (c 0.05, CHCI_3_); Yield: 25 mg, yellow oil. IR (KBr, cm^−1^): 3448, 3018, 2359, 2339, 2099, 1733, 1637, 1452, 1368, 1283, 1245, 1219, 1178, 1096, 1025. ^1^H NMR (400 MHz, CDCl_3_, δ in ppm) δ = 5.94 (1H, d *J*= 4.1 Hz, H-1), 5.66 (1H, m, H-2), 5.57 (s, H-6), 5.02 (1H, d, *J*= 2.2 Hz, H-3), 5.0 (1H, d *J*= 6.8 Hz H-9), 2.63–2.59 (1H, m, H-7), 1.44 (1H, m, H-15), 1.98 (3H, s, H-12), 1.99, (3H, s, H-13), 1.6, (3H, s, H-14), 2.10, (3H, s), 2.16, (3H, s), 2.24 (3H, s), 3.93, (1H, s), 8.16–7.89 (5H, m), 7.61–7.37 (5H, m); ^13 ^C NMR (100 MHz, in ppm). δ = 90.28 (C-5), 81.13 (C-11), 74.81 (C-10), 74.66 (C-3), 72.27 (C-9),72.09 (C-1, 69.72 (C-4), 68.97 (C-2), 50.98 (C-10), 50.24 (C-7), 30.38 (C-12), 21.36 (C-13), 16.40 (C-15), 30.38 (C-8), 24.64 (C-14), 81.12 (C-6). HRMS (ESI, *m/z*): calculated for C_35_H_40_O_7_ [M + H] ^+^ 573.6879 found 573.6875.

*Compound* ***12****; 1α,2α,3α,8α,14-pentaacetoxy-6β,9β-dibenzyloxy-4β-hydroxidihydro-β-agarofuran*: Yield: %, Yield: 25 mg, yellow oil; IR (KBr, cm^−1^):3428, 3008, 2319, 2139, 2099, 1730, 1645, 1341, 1258, 1236, 1153, 1006. ^1^H NMR (400 MHz, CDCl_3_, δ in ppm) δ = 5.68–5.64 (1H, m, H-2), 5.17 (1H, d, *J*= 4.0 Hz, H-9), 5.13 (1H, s, H-8), 4.96–4.95 (1H, m, H-3), 4.92 (1H, d, *J*= 2.1 Hz, H-1), 4.83 (1H, d, *J*= 2.1 Hz, H-6), 4.72–4.68 (1H, m, H-15), 1.44, (1H, d, *J*= 6.3 Hz, H-4), 1.4 (3H, s, H-13), 1.38 (3H, s, H-12), 3.93 (1H, s, OH-4), 8.12–8.03 (5H, m, 6-OBz), 2.16 (3H, s, 1-OAc), 2.11 (3H, s), 2.08 (3H, s), 1.97 (3H, s), 1.92 (3H, s); ^13 ^C NMR (100 MHz, in ppm). δ = 91.70 (C-5), 82.62 (C-11), 71.45 (C-4), 73.00 (C-3), 72.6 (C-6), 72.5 (C-9), 71.6 (C-8), 70.15 (C-1), 69.57 (C-2), 61.14 (C-15), 50.75 (C-10), 54.81 (C-7), 24.4 (C-14), 26.41 (C-12), 26.18 (C-13). HRMS (ESI, *m/z*): calculated for C_39_H_44_O_9_ [M + H] ^+^ 657.3019 found 657.3015

*Compound* ***13****; 6β-acetoxy-9β-benzyloxy-1α,8α-dihydroxydihydro-β-agarofuran*: [a] _D_^20^ −29.0 (MeOH, c l0.0), Yield: 25 mg, yellow oil. IR (KBr, cm^−1^): 3420, 1720, 1280, 710. ^1^H NMR (400 MHz, CDCl_3_, δ in ppm) δ = 5.97 (1H, d, *J*= 7.1 Hz H-6), 5.12 (1H, m, H-9), 4.68 (1H, dd, *J*= 12.0, *J*= 4.3 Hz H-8), 3.61 (1H, m, 3.72–3.47 H-1), 2.51 (1H, m, 2.54–2.47 H-7), 2.10 (3H, s, 6-OAC), 1.8 (1H, m, H-4), 1.49 (3H, s, H-15), 1.41 (3H, s, H-13), 1.37 (3H, s, H-12), 0.96 (3H, d, *J*= 7.4 Hz, H-14), 8.10 (5H, dd, *J*= 1.0, *J*= 7.1 Hz, 9-OBZ); ^13 ^C NMR (100 MHz, in ppm). δ = 89.76 (C-5), 82.57 (C-11), 79.16 (C-9), 72.44 (C-6), 68.30 (C-1), 55.79 (C-7), 55.78 (C-10), 33.78 (C-4), 27.10 (C-2), 30.28 (C-12), 30.45 (C-13), 26.23 (C-3), 16.66 (C-15), 17.08 (C-14), 72.45 (C-8). HRMS (ESI, *m/z*): calculated for C_24_H_32_O_5_ [M + H] ^+^ 401.2283 found 401.2279.

### 3.6. *In vitro* AChE/BChE inhibitory activity assay

The evaluation of enzyme inhibition was carried out using an adapted version of Ellman’s method^21^ in the 96-well plates. AChE (from *Electrophorus electricus*) and BChE (from equine serum), 5,5′-dithio-bis-(2-nitrobenzoic acid (DTNB), acetylthiocholine and butyrylthiocholine iodides were purchased from Sigma-Aldrich. The stock solutions of the test compounds were prepared in 100 µL of DMSO and 900 µL of phosphate buffer (8 mmol/L K_2_HPO_4_, 2.3 mmol/L NaH_2_PO_4_, 150 mmol/L NaCl, and 0.05% Tween 20 at pH 7.6). Enzyme solutions were prepared with buffer to give 0.25 U/mL and 50 µL was added to the plate. After 30 min of incubation, the substrate solution consisting of Na_2_HPO_4_ (40 mmol/L), acetylthiocholine/butyrylthiocholine (0.24 mmol/L) and DTNB (0.2 mmol/L) was added. Absorbance of the yellow anion product - due to the spontaneous substrate hydrolysis - was measured at 405 nm for 5 min on a Microliter plate reader (Epoch, Biotek). The enzyme activity was calculated as a percentage compared with a control using only the buffer and enzyme solution. The compounds were assayed in a dilution interval of 500–1.3 µg/mL. Galantamine and carvacrol was used as a positive control.

### 3.7. Kinetic characterisation of AChE inhibition

The kinetic characterisation of AChE was performed using a previously reported method^25^. The enzyme solution was preincubated with different substrate concentrations ranging from 9.38 × 10^−4^ to 0.48 mM. Compound **4**, used like as inhibitor was added to the test solution at five different concentrations and the experiment was performed in triplicate. Data analysis was carried out with SigmaPlot v10.0.

### 3.8. COX inhibitory activity

Synthesised compounds and the positive control drugs, Celecoxib, Diclofenac sodium and Indomethacin, were tested for their ability to inhibit COX-1 and COX-2 using COX Inhibitor Screening Assay kit (ACE™ EIA kits, catalog no. 560131). The assay includes both ovine COX-1 and human recombinant COX-2 enzymes. Various concentrations for each of the tested compounds’ stock solutions (0.5, 1, 2.5, 5 and 10 mM) were used to obtain the concentration inhibition response curve and calculate the concentration of the test compound causing 50% inhibition (IC_50_, mM).

### 3.9. Ligand Construction and molecular docking studies of compounds with AChE and COX-1/2

All structures of ligands were constructed using Spartan'10 1.1.0 2011 and their geometries were optimised using Density Functional B3LYP 6–31**G ab initio methods in a vacuum. These studies were performed using X-ray crystal structure of *T. californica* (PDB 1EA5; 1, 8 Å resolution)^40^ acetylcholinesterase, the crystal structure of COX-1 enzyme with 2.6 Å resolution complex (PDB code: 3N8Y) and COX-2 enzyme with 1.73 Å resolution complex (PDB code: 3NT1) was obtained from the PDB database https://www.rcsb.org/. Docking molecular of ligands **3**–**13** were carried out with the AUTODOCK 4 package using the Lamarckian genetic algorithm^41^. After the preparation of the autodock format of protein and selected compounds, the obtained enzyme structure was used as an input for the AUTOGRID program.

AUTOGRID for each atom type in the ligand performed a precalculated atomic affinity grid map, plus an electrostatics map and a separate desolation map presented in the ligand. The centre of the grid box to AChE was placed in coordinates x = 3.436, y = 63.459, z = 66.849. The dimensions of the active site box were set at 112 × 126 × 122 points. All maps were calculated with 0.236 Å spacing between the grid points. The parameters were the following: a grid box was prepared individually for COX-2 to cover the pocket with the main residues for enzyme binding site by maintaining the grid size at 82 × 70 × 62 Å in the x-, y-, and z-axis, respectively, and a grid size of x = 80, y = 70, z = 58 Å for the COX-1 enzyme, centred at coordinate x, y, z −33.802, −45.341, −24.413 (for COX-2) and 36.007, −52.132, −1.553 (for COX-1) respectively, with 1 Å spacing between the grid points^42^. Searching parameters were to 50 runs and with 25.000.000 maximum number of evaluations for each ligand. All experiment was making a physiological pH. Root-mean-square deviation (RMSD) between the best redocked conformation and the original conformation of the ligand was then calculated using the PyMOL software. The best pose of each ligand was selected for analysing the interactions between enzyme and the inhibitor. The results were visualised using Discovery Studio 4.0 Client.

### 3.10. Propidium displacement studies

The ability of compounds to competitively displace propidium iodide was evaluated by a fluorescence method^43^. To determine the degree of displacement (% displacement) of propidium iodide from the PAS of AChE, EeAChE (fnal concentration 7 µM) was incubated with the test compound at a concentration of 290 and 340 µM in 1 mM Tris-HCl bufer pH 8.0, 25 °C for 15 min. Then, propidium iodide solution (final concentration 8 µM) was added, the samples were incubated for 15 min and the fluorescence spectrum 530 nm (excitation) and 600 nm (emission) was taken. Donepezil was used as reference compound. The blank contained propidium iodide of the same concentration in 1 mM Tris-HCl buffer pH 8.0. The measurements were carried out in triplicate on a microplate reader Perkin Elmer VictorX2 (Perkin Elmer, Singapur).

## 4. Conclusion

In conclusion, we either isolate or synthesise a group of molecules with dihydro-β-agarofuran skeleton (epoxieudesmanes) and evaluated their potential as inhibitors of the enzyme acetylcholinesterase, butyrylcholinesterase, COX-1 and COX-2. The structures of the compounds were characterised in the basis of NMR, IR, and MS. Both, synthetic and natural compounds showed inhibitory activity against the acetylcholinesterase enzyme of *Torpedo californica* with IC_50_ values ranging from 17.0 ± 0.016 − 740.0 ± 0.045 µM. Compound **4** has the best inhibitory activity of the enzyme AChE. The compounds evaluated showed selectivity for AChE, with inhibitory activity on the BChE enzyme observed only at high concentrations.

Regarding the COX-1 and COX-2 enzymes, we can say that the compounds act as inhibitors of both isoforms. Compound **9** (IC_50_ 8.69 µM) showed the best inhibitory activity on COX-1, while compound **8** (IC_50_ 0.04 µM) showed the best inhibitory activity on COX-2. Natural compounds are better inhibitors than synthetic compounds, in addition the compounds exhibited a preference for the COX-2 isoform.

The study of molecular docking and enzymatic kinetics showed that the sesquiterpenes with dihydro-β-agarofuran skeleton inhibit the AChE enzyme, through a non-competitive mechanism. Moreover, our data suggest that the inhibition is due to different interactions with the peripheral anionic site (PAS). Among the forces involved in these interactions it could be mention Van der Waals attraction and Pi-sigma, Pi-alkyl and hydrogen bonds formation. Binding of the compounds to the AChE PAS together with the type of AChE inhibition suggests their potential to block the aggregation of β-amyloid induced by AChE. In fact, the compounds studied effectively displaced the propidium of AChE PAS.

Molecular docking studies of COX isoforms show that compounds interact with these enzymes through conventional and unconventional hydrogen bonds, stabilising the compounds with the protein, transforming them into a potential source of new anti-inflammatory compounds that could be explored in the future as a cure for the many neurodegenerative diseases mediated by neuroinflammation.

Finally, based on druglikeness studies, ADMET and related properties, it was determined that the evaluated molecules had good pharmacokinetic properties. When the results were evaluated in terms of in silico and *in vitro* analysis, good agreement was achieved between experimental and theoretical results. In summary, this study provided new small molecules of AChE and COX inhibitors with good therapeutic potential for neurological disorders such as Alzheimer's disease and other causes of dementia.
